# Treatment of hypothyroidism improves glomerular filtration rate (GFR) in geriatric patients

**DOI:** 10.3906/sag-2011-257

**Published:** 2021-06-28

**Authors:** Mert EŞME, Oktay BULUR, Mehmet Can ATAK, Cafer BALCI, Kürşat DAL, Derun Taner ERTUĞRUL, Zeliha Günnur DİKMEN, Meltem Gülhan HALİL, Mustafa CANKURTARAN, Burcu BALAM DOĞU

**Affiliations:** 1 Division of Geriatric Medicine, Department of Internal Medicine, Faculty of Medicine, Hacettepe University, Ankara Turkey; 2 Department of Internal Medicine, Keçiören Training and Research Hospital, Ankara Turkey; 3 Department of Internal Medicine, Faculty of Medicine, Hacettepe University, Ankara Turkey; 4 Department of Medical Biochemistry, Faculty of Medicine, Hacettepe University, Ankara Turkey

**Keywords:** Hypothyroidism, older, renal

## Abstract

**Background/aim:**

Renal function of patients with hypothyroidism increases after reaching euthyroid state. There is no data regarding geriatric age group. The aim of the study was determined to investigate whether renal function of geriatric patients with hypothyroidism increases after they become euthyroid.

**Materials and methods:**

Patients who were sixty-five years or older were retrospectively screened in two centers. TSH, T3, T4, creatinine, and eGFR calculated by CKD-EPI formula were recorded under the presence of accompanying hypothyroidism. The same variables were recorded after the patients became euthyroid.

**Results:**

285 patients were included in the study, the median age was 73(65–84), and 234 patients were female. Patients were examined in four groups according to TSH values. There were 160 (56.1%) patients with TSH 5–9.9 uIU/mL, 60(21.1%) patients with TSH between 10–19.9 uIU/mL, 41(14.4 %) patients with TSH between 20–49.9 uIU/mL and 24(8.4%) patients with TSH> 50uIU/mL. There was a significant and negative correlation between the initial TSH values and the first calculated eGFR values (p: 0.001; r: –0.191). The median eGFR of the patients in hypothyroid cases was 66.59 (14.62–116.07), while the median eGFR value of patients was 69.6 (12.91–109.31) in the euthyroid state. This value obtained after thyroid replacement was significantly improved when compared to the first eGFR (p: 0.001). In logistic regression analysis, pretreatment TSH value was found to independently affect eGFR (p: 0.009; Exb: 1.017).

**Conclusion:**

It has been observed that hypothyroidism treatment increases eGFR in geriatric patients. Similar results were obtained after studies with younger patients in the literature. This study is a study in which only geriatric age group patients were examined. It should be kept in mind that hypothyroidism which is not corrected in geriatric patients may also contribute to a decrease in eGFR.

## 1. Introduction

Thyroid hormone interacts with all systems. In addition to its effect on all systems, it plays a role in the growth and development of the kidneys. It also regulates water and sodium balance [1]. Due to its effect on the kidney, it also regulates renal plasma flow (RPF) and glomerular filtration rate (eGFR). Hyponatremia with decreased RPF and eGFR are common in patients with hypothyroidism. It has been observed that these disorders improve with thyroid replacement therapy [2]. It has been shown in the literature that L-thyroxine can correct acute renal failure and that underlying chronic renal failure rapidly worsens in patients with hypothyroidism [3,4].

Thyroid disorders affect all systems, primarily the cardiovascular and renal systems [5]. The renin-angiotensin system (RAS) functions to control cardiovascular and renal function. Thyroid hormones play an important role in the growth and development of various tissues, including kidney and lung, which are the main sites of renin and ACE synthesis.

Hypothyroidism is a disease that is more common in older people than young people and is more common among women than men. The incidence increases with age. In a study that evaluated more than 25,000 people, it was found that 10% of men aged 65 to 74 and 16% of women had TSH levels above the normal limit. It was observed that TSH levels increased in 16% of men aged 75 and over, and 21% of women [6]. In the latest National Health & Nutrition Examination Survey (NHANES) study, age-specific analysis of measured TSH levels and antithyroid antibody titers, without evidence of autoimmune thyroiditis, showed that 12% of subjects aged 80 and over had TSH levels greater than 4.5 mIU/L [7].

Old age is considered to be a risk factor for chronic kidney disease (CKD), but the decrease in the eGFR with aging may be a consequence of the normal aging process and not a sign of disease. 

Although correction of hypothyroidism has been shown to have a positive effect on eGFR [8], no previous studies have been conducted in older patients. In this study, it was aimed to examine the change in kidney functions of older patients with hypothyroidism after they became euthyroid.

## 2. Materials and methods 

### 2.1. Study subjects

This study was conducted in two centers, one of which was a university hospital and the other was a government hospital. In the last 10 years, 1151 patients who were admitted to Geriatric Medicine and Endocrinology outpatient clinics with hypothyroidism diagnosis were retrospectively examined. Patients under 65 years of age and patients without TSH and creatinine values were excluded from the study. As a result, the results of 285 patients were examined. The local ethics committee approved the study. 

### 2.2. Laboratory tests

Free T3 (fT3), free T4 (fT4), TSH, creatinine, and eGFR measurements were examined in both hypothyroid and euthyroid states. Although it changed according to the initial TSH values of the patients, The duration of TSH groups to become euthyroid state is between 10–14 weeks. For patients who became euthyroid, the normal TSH value was determined as the value within the normal reference range of the laboratory. The eGFR was calculated using the chronic kidney disease epidemiology collaboration equation (CKD-EPI). The CKD-EPI equation was developed to find a more precise formula than serum creatinine and other preset clinical parameters when the eGFR is > 60 mL/min per 1.73 m2. The CKD-EPI equation performed better than the MDRD (modification of diet in renal disease study) equation.

The CKD-EPI equation:

eGFR = 141 * min(Scr/κ,1)α * max(Scr/κ, 1) –1.209 * 0.993 age * 1.018 [if female] * 1.159 [if black] (Scr is serum creatinine (mg/dL), κ is 0.7 for females and 0.9 for males, α is –0.329 for females and –0.411 for males), min indicates the minimum of Scr/κ or 1, and max indicates the maximum of Scr/κ or 1) [9].

Serum levels of fT3 (normal 3.8–6 pmol/L), fT4 (normal 7.86–14.41 pmol/L) and TSH (normal 0.38–5.33 mIU/mL) were measured by a paramagnetic particle chemiluminescent immunoassay using the UniCel DxI 800 Immunoassay System (Beckman Coulter, USA). Serum creatinine was measured by Jaffe method at AU 5800 (Beckman Coulter, USA).

### 2.3. Statistical analyses

Distribution of the continuous variables was determined by the Kolmogorov–Smirnov test. Continuous variables with normal distribution were expressed as mean ± SD. Variables with skew distribution were expressed as median (minimum-maximum) and categorical variables were expressed as percentage. The paired sample t-test was used for normally distributed variables. The Wilcoxon test was used to compare dependent variables that were not normally distributed. Pearson and Spearman analysis was used to identify correlations between study parameters. To compare the parameters which were not normally distributed the Kruskal–Wallis tests were conducted. The Mann–Whitney U test was performed to test the significance of pairwise differences using Bonferroni correction to adjust for multiple comparisons. Linear regression analysis and logistic regression analysis were statistical methods used to find factors that independently affect glomerular filtration rate. For all statistics, a two-sided p value < 0.05 was considered to be statistically significant. All analyses were performed with SPSS v: 22.0 for Windows (SPSS Inc, Chicago, IL).

## 3. Results

285 patients were included in our study. The median age was 73 (65–94) years. Patients were divided into four groups according to their TSH values. There were 160 patients (56.1%) with TSH value between 5–9.9 μIU/mL, 60 patients (21.1%) with a TSH value between 10–19.9 μIU, 41 patients (14.4%) with a TSH value between 20–49.9 μIU/mL and 24 patients (8.4%) with TSH >50 μIU/mL. 234 patients were female and 51 were male. Demographic characteristics are shown in Table 1. There was a statistically significant negative correlation between the TSH value in the baseline hypothyroid phase and the eGFR values at that time (p: 0.001; r: –0.191). Furthermore, there was a significant and negative correlation between the TSH levels and the eGFR values at the time patients became euthyroid (p: 0.016; r: –0.155). The median eGFR of the patients with hypothyroid status was 66.59 (14.62–116.07), while the median eGFR value of the patients with euthyroid status was 69.6 (12.91–109.31). The difference between these values after thyroid replacement was statistically significant (p: 0.001). These differences are shown in Table 2. The difference between TSH values at baseline and at the end of treatment was also significant (p: 0.001).

**Table 1:** Demographic features.

	Age	Center 1	Center 2	TSH Group 1	TSH Group 2	TSH Group 3	TSH Group 4	Total
Age		73(65–89)	74(65–94)	74(65–94)	73(65–93)	72(65–85)	72.5(65–86)	
Male	73(66–93)	148	22	22	14	8	7	51
Female	73(65–94)	29	86	138	46	33	17	234
Total	73(65–94)	177	108	160	60	41	24	285

**Table 2:** eGFR and TSH changes before and after treatment.

	Before treatment	After treatment	P value
eGFR	66.59(14.62–116.07) mL/dk	69.6(12.91–109.31) mL/dk	0.001
TSH	18.15 ± 22.39 uIU/mL	2.6 ± 1.5 uIU/mL	0.001

Initially, patients were divided into 4 different groups according to TSH values. There was a significant difference between these groups in terms of eGFR values by the Kruskal–Wallis test (0.017). Post-hoc analyses were performed to determine which group lead to this significance. The difference between the groups analyzed by Mann–Whitney U test after post-hoc analysis was considered significant if the p-value was below 0.008. The results revealed that the difference was caused by the difference between the first and fourth groups of TSH values and the second and fourth groups of TSH values (p: 0.006; p: 0.005, respectively). These significant differences are shown in Table 3. eGFR changes in four TSH groups before and after treatment were examined. eGFR change in the 4th group was highest. Similarly, whether the change in eGFR before and after treatment was different between TSH groups was examined with the Kruskal–Wallis test. There was a statistically significant difference between the groups (p: 0.001). It was seen that this difference originated from the 4th group (p: 0.001). The median eGFR change in group 4 was 12.47 mL/min/m2. The graph of the change between TSH groups is shown in Figure.

**Table 3:** eGFR differences between groups before treatment.

TSH groups	eGFR	P value : 0.017
1	69.27 (15.02–166.07) mL/dk	
2	68.88 (14.62–110.8) mL/dk	
3	64.82 (29.20–90.13) mL/dk	
4	57.43 (22.45–94.96) mL/dk	

**Figure:**
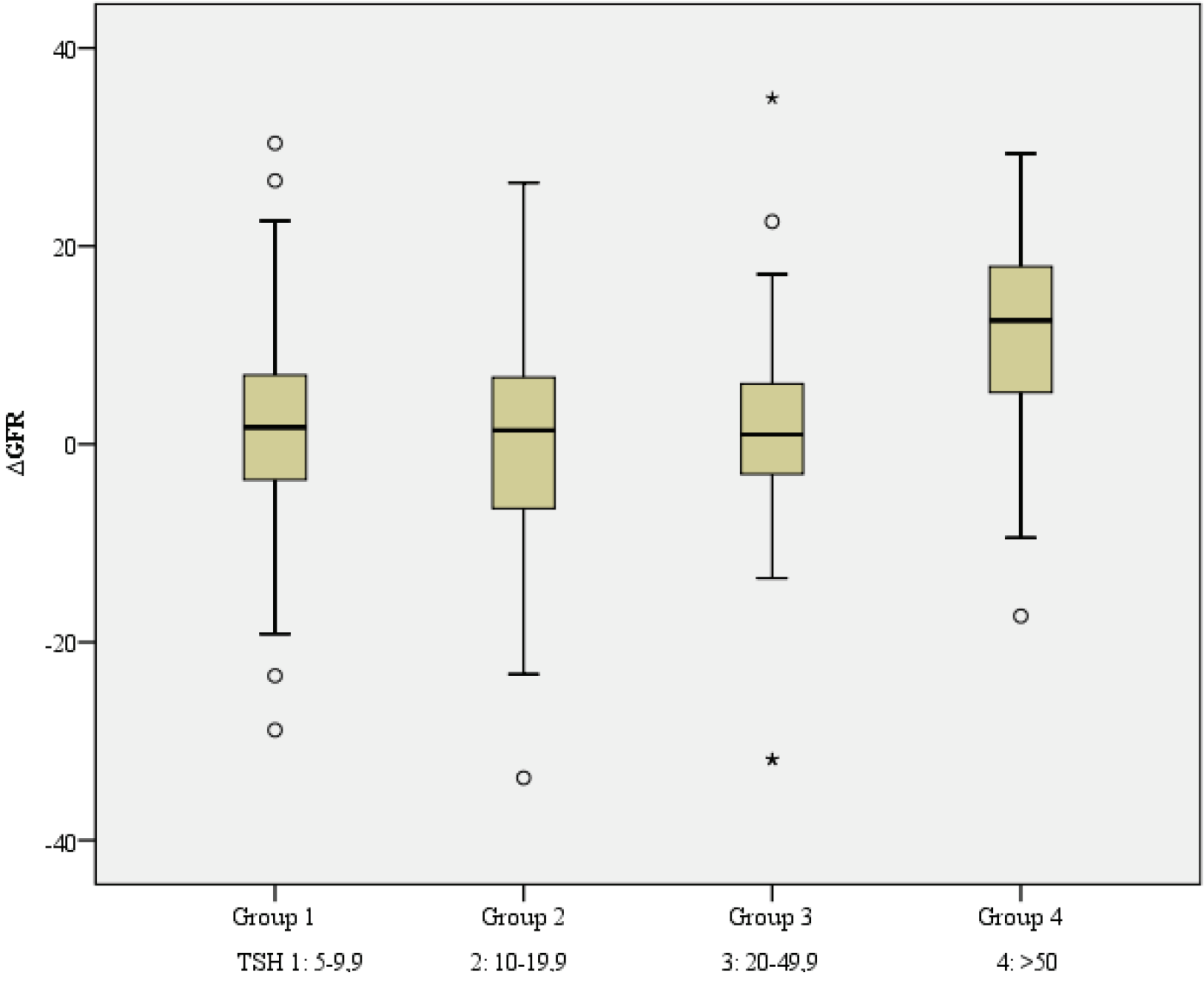
eGFR changes of patients after treatment of according to TSH groups.

When the factors affecting eGFR changes before and after treatment were wanted to be examined by linear regression analysis, age, pre-treatment TSH value and post-treatment TSH value were included in the model. It was observed that the increase in eGFR was higher in patients with higher TSH values before thyroid replacement therapy (p: 0.001). Similarly, when patients with and without increased eGFR before and after treatment were dichotomized and logistic regression analysis was performed, it was observed that there was an increase in eGFR in patients with high TSH values before treatment (p: 0.009; Exb: 1.017). Regression analyzes are shown in Table 4.

**Table 4:** Factors affecting eGFR changes.

Model 1* linear regression analysis	P value	Beta	95% CI
eGFR change			
Age	0.497	0.040	–0.111–0.229
Pretreatment TSH	0.001	0.209	0.045–0.154
Posttreatment TSH	0.697	0.023	–0.649–0.969
The model fit was assessed using appropriate residual and goodness-of-fit statistics. (F sig : 0.001)			
Model 2** logistic regression analysis		ExB	
eGFR change			
Age	0.312	1.017	0.984–1.051
Pretreatment TSH	0.009	1.017	1.004–1.031
Posttreatment TSH	0.746	0.260	0.876–1.204
Hosmer and Lemeshow goodness of fit statistics were used to assess model fit (p: 0.086).			

## 4. Discussion

In this study, we wanted to evaluate the effect of hypothyroidism on renal functions in older patients. The results of this study revealed an improvement in renal function and an increase in GFR after correcting thyroid functions. The group with the greatest improvement in eGFR after becoming euthyroid was the group with the highest baseline TSH levels (TSH > 50 μIU/mL) at baseline. In the literature, there are studies with heterogeneous patient groups examining the effects of thyroid replacement therapy on renal functions. Our study is important because this is examining only the geriatric patient group. Due to changes in the renal function of the older patients, this issue needs to be studied in the older group. This study fills this gap in the literature and demonstrates that the improvement in thyroid functions also improves renal functions.

The results in the literature suggest that the subclinical form of hypothyroidism may also be associated with decreased eGFR and a high prevalence of chronic kidney disease [10–14]. The results of this present study support these observations and suggest that low thyroid function within the clinically normal range is associated with reduced GFR. In addition, thyroid function, which is within normal limits, is positively associated with effective renal plasma flow, a measure of renal blood flow [13,15]. After treatment of hypothyroidism with T4 therapy, increased renal blood flow was observed. Thus, renal blood flow may mediate the relationship between thyroid function and GFR. Since high vascular resistance and low cardiac output are the vascular consequences of hypothyroidism, a decrease in renal blood flow may ocur [2,16]. On the other hand, it is also possible for kidney function to affect the thyroid. Therefore, renal dysfunction may cause elevated serum iodine levels and observations in patients with end-stage renal disease suggest that iodine restriction may improve hypothyroidism in these patients [2]. There are studies suggesting that autoimmune thyroid disease may lead to immunocomplex accumulation in renal glomeruli [2]. In our study, autoantibody levels of patients were not determined. Therefore, we cannot make an inference to support this information.

Histological changes in nephrons, especially basement membrane thickening, have been demonstrated in both hypothyroid rats and humans. These changes can lead to changes in renal hemodynamics, reductions in renal blood flow, physiological effects that reduce GFR and, therefore, creatinine clearance [17–19]. 

Lo et al. investigated the prevalence of hypothyroidism at different levels of predicted GFR. In this study, it was found that low GFR was associated with a higher prevalence of hypothyroidism [20]. Shin et al. evaluated the effect of LT4 treatment on stage 2–4 CKD patients, suggesting that LT4 decreases renal function decline in CKD patients with subclinical hypothyroidism [21]. Arora et al. Found that 46 patients with hypothyroidism had significantly reduced serum creatinine levels in response to 6 weeks of treatment with thyroxine [22]. In our study, there were 30 patients with creatinine levels above normal, and 9 of them showed a significant decrease in creatinine levels after treatment.

The RAS system may be one of the factors explaining renal recovery from LT4 replacement. It is known that thyroid hormones stimulate renin mRNA through mechanisms dependent on the renin hormone regulatory element [23]. Thyroid hormone can also affect tubular functions. T3 increases the expression of mRNA encoding Na+/K+-ATPase. For this reason, hyponatremia may be seen due to increased sodium urinary excretion. It is therefore important to distinguish TSH values when making the differential diagnosis of hyponatremia.

The number of patients in our study is sufficient to make an adequate inference. Some limitations of this study should be addressed. Since this was a retrospective study, a screening was performed on the hospital records. There are many factors that may impair renal function in older patients. The first of these are the reasons for not getting enough fluid, using multiple NSAIDs, antihypertensive drugs, diuretics, prostate problems in male patients. In our database, there was no record of the patients’ possible problems. For this reason, we could not observe adequate recovery after thyroid replacement in some patients. In some patients, creatinine levels increased and GFR values decreased. It was thought that the reason for this is not known whether or not the causes of possible renal dysfunction are mentioned above. However, we cannot ignore the possibility of hypothyroidism causing glomerulosclerosis and permanently impairing kidney function, as shown by studies. As a result of our study, we cannot say for sure that hypothyroidism may lead to direct renal injury in geriatric patients, but it is clear that there is a correlation between GFR and thyroid functions. Further prospective studies are needed to make clear conclusions regarding this issue.

As a conclusion, to the best of our knowledge, this study is important in that it is a study in which only geriatric patients were examined and showed that renal functions could improve after hypothyroidism treatment. The high significance of the results and the large study population are the strengths of this study. It should be kept in mind that decreased renal function and thyroid disorders may have a link in older age. Screening TSH levels should be a routine part of geriatric assessment. Moreover, renal function of geriatric patients should be re-assessed after giving hypothyroidism treatment and achieving euthyroid state. 

## Informed consent

Ethics committee approval was obtained for the study. The committee from whom received ethical approval, did not deem the patient’s informed consent necessary since the study was retrospective.
